# Contra Indications of Iron

**Published:** 1878-05

**Authors:** 


Contra-Indication of Iron.—There are two different states found in women where iron is either totally contra-indicated or to be given with great caution. The first is a condition of amenorrhœa in florid, plethoric persons. The other is the opposite condition of menorrhagia in certain females. There are cases of menorrhagia associated with pallor and debility, where the usual compound of iron and extract of ergot is not so useful as a non-chalybeate treatment. In these cases it is not any imperfection in the process of blood manufacture which is to be remedied, for the blood is made rapidly and quickly, only to be lost at each menstrual period. It is here desirable rather to limit the rapidity of the blood formation, so that when the several vascular turgescence of the menstrual period comes, it will not find the blood vessels too distended with blood. This will lead to diminished catamenial loss, and so the blood waste will be economized. According to the experience of Dr. Brown Séquard and Dr. Hughlings Jackson, iron does not suit epileptics. It increases the tendency to fits. It may improve the general condition, but it aggravates the epilepsy.—(*Dublin Medical Press*, Oct. 3.)—*Chicago Med. Jour. & Ex.*

